# A non-randomized clinical trial to evaluate the effect of fingolimod on expanded disability status scale score and number of relapses in relapsing-remitting multiple sclerosis patients

**DOI:** 10.1186/s40169-019-0228-7

**Published:** 2019-04-08

**Authors:** Mehrdokht Mazdeh, Shahriar Kargar Monhaser, Mohammad Taheri, Soudeh Ghafouri-Fard

**Affiliations:** 1 Department of Neurology, Hamadan University of Medical Sciences, Hamadan, Iran; 2 Neurophysiology Research Center, Hamadan University of Medical Sciences, Hamadan, Iran; 3 Urogenital Stem Cell Research Center, Shahid Beheshti University of Medical Sciences, Tehran, Iran; 4 Department of Medical Genetics, Shahid Beheshti University of Medical Sciences, Tehran, Iran

**Keywords:** Fingolimod, EDSS score, Multiple sclerosis, Relapse

## Abstract

**Background:**

Multiple sclerosis (MS) is a chronic disease characterized by demyelination, glial activation and axonal degeneration in the central nervous system. At the present, there is no certain remedy for this disease. However, available therapies often attenuate disease progress.

**Methods:**

This study aims at identification of the effect of fingolimod on expanded disability status scale (EDSS) score and number of relapses in relapsing-remitting MS (RRMS) patients in comparison with IFNβ. In the present 12-month non-randomized clinical trial, 55 RRMS patients aged between 18 and 45 with EDSS scores between 0 and 5.5 were divided into two groups. Twenty-five patients received 0.5 mg oral fingolimod once a day for 12 months and 30 patients were under treatment with IFNβ. EDSS scores and number of relapses were recorded for all study participants monthly.

**Results:**

No significant difference was found in age and sex of patients recruited in two study groups. EDSS score was significantly lower in treatment group in month 10, 11 and 12 after treatment compared with control group (p values of 0.004, 0.006 and 0.007 respectively).

**Conclusion:**

Treated patients experienced no relapse during the study period. Fingolimod is effective in reduction of EDSS score and number of relapses in Iranian MS patients.

## Introduction

Multiple sclerosis (MS) is a chronic disorder of the central nervous system characterized by demolition of myelin sheath, glial activation and axonal degeneration [1]. Several treatment strategies have been suggested for this disorder among them is fingolimod. Fingolimod is approved as a second-line therapeutic option in the Europe and as first-line in the United States, Canada and other regions [2]. Fingolimod is a small lipophilic substance with a sphingosine-like configuration, which binds with the sphingosine-1-phosphate (S1P) receptor family members on the surface of lymphocytes and suppresses lymphocytes marshaling to the peripheral blood. Retention of central memory T-cells, T_H_17 cells, and B-cells in the peripheral lymphoid tissue considerably decreases contact of autoreactive lymphocytes with the central nervous system (CNS), therefore moderating the inflammatory response in MS patients [3]. Moreover, fingolimod interaction with S1PR1, S1PR3, and S1PR5 on the surface of neurons, astrocytes, oligodendrocytes, and microglia results in neuroprotective and regenerative processes including neuronal damage healing, increase of oligodendrocytes survival, enhancement of oligodendrocytes progenitors quantity and remyelination as revealed cell culture and animal models [4]. Being administered as a single-daily capsule, the efficacy of this drug has been promising in MS [3]. Regardless of some safety issues, the efficacy of fingolimod on evolution of disability in MS patients has been compared with placebo or interferon β (IFNβ) in the two pivotal Phase III trials [5]. Although the placebo-controlled trial reported improvement of relapse rate and risk of disability progression [6], in the second trial no data on EDSS were clearly commented [5].

We conducted the present non-randomized clinical trial to assess the effectiveness of fingolimod on reduction of disability and relapse rate in Iranian patients with relapsing-remitting MS (RRMS) in comparison with IFNβ.

## Methods

The present study was a 12-month non-randomized clinical trial assessing the efficacy of once-daily fingolimod (0.5 mg) on relapse rate and expanded disability status scale (EDSS) score of RRMS patients in comparison with IFNβ. Patients were referred to Farshchian Hospital, Hamadan, Iran during 2015–2016. The study protocol was approved by the ethical committee of Hamadan University of Medical Sciences. Informed consent forms were obtained from all study participants. Availability sampling method was used. Sample size was estimated to be 60 (30 patients in fingolimod-treatment group and 30 IFNβ-treated patients as controls) based on the parameters obtained from previous studies [5]:

D = µ1 − µ2 = 0.3, α = 0.05, β = 20%, power = 80%, µ1 (relapse rate in control group) = 0.4, µ2 (relapse rate in fingolimod treated group) = 0.1

Five patients in the treatment group left the study due to personal reasons. Demographic data, disease duration, EDSS score and previous treatment strategies were recorded from all study participants. The inclusion criteria for treatment group were RRMS based on the revised McDonald criteria [7], age between 18 and 45, one or more confirmed relapses during the prior year, EDSS score of 0–5.5, intolerance to IFNβ therapy and no relapse or steroid treatment within 30 days before study initiation. Complete blood count, liver function test and Varicella-Zoster immune status were tested in all patients. Patients with primary or secondary progressive MS, history of other chronic disorders, malignancy, pregnancy, macular edema, active bacterial/viral/fungal infection, previous administration of cyclophosphamide/mitoxantrone/monoclonal antibodies, chronic liver disease, elevation of liver enzymes/bilirubin/alkaline phosphatase/creatinine, white blood cell < 3500 or lymphocyte count < 800 were excluded from the study. Patients were evaluated for bradycardia by hourly assessments of pulse and blood pressure for 6 h after fingolimod administration. In addition, an electrocardiogram (ECG) prior was obtained before administration of the first dose and at the end of the study period.

Control group consisted of patients who were under treatment with IFNβ (intramuscular injection of 20 μg of CinnoVex [CinnaGen Co, Tehran, Iran] three-times a week). The inclusion criteria for control group were RRMS based on the revised McDonald criteria [7], age between 18 and 45, one or more confirmed relapses during the prior year, EDSS score of 0–5.5 and no relapse within 30 days before study initiation. Patients with primary or secondary progressive MS disorder, chronic liver or thyroid disorder or elevated alkaline phosphatase levels were excluded.

Data were analyzed using SPSS version 16. The Kolmogorov–Smirnov test was used for assessment of data distribution. Relapse rate and EDSS score mean values before and after treatment were compared using paired T test and Wilcoxon test based on the normality of data. These values were compared between two study groups using dependent T and Mann–Whitney tests based on the normality of data. Chi square test was used for assessment of association between categorical variables. The relationships between quantitative variables were evaluated using Pearson or ANOVA tests. P values less than 0.05 were considered as significant. EDSS scores in each month were compared using ANOVA test.

## Results

There was no significant difference in age and sex ratio between two study groups (Treatment group (Mean age ± SD): 39.08 ± 7.63, Control group (Mean age ± SD): 36.83 ± 7.24, P = 0.269; female/male ratio of 24/6 and 18/7 respectively, P = 0.487). Table 1 summarizes the demographic and clinical data.

**Table 1:** The demographic and clinical data

Parameters	Fingolimod-treatment group	IFNβ-treatment group
Age (mean ± SD)	39.08 ± 7.63	36.83 ± 7.24
Sex ratio (Female/male)	24/6	18/7
Disease duration	8.20 ± 3.42	8.77 ± 2.5
EDSS (mean ± SD)	4.92 ± 0.73	4.52 ± 1.09

The clinical manifestations of disease in each study group are demonstrated in Table 2.

**Table 2:** The clinical manifestations of disease in each study group

Clinical symptoms and signs	Treatment group Number (%)	Control group Number (%)
Blurred vision	3 (10)	8 (32)
Diplopia	6 (20)	4 (16)
Gait problem	4 (13.3)	2 (8)
Paresthesia	8 (26.6)	6 (24)
Muscle weakness	3 (10)	3 (12)
Dysarthria	0 (0)	6 (24)
Bladder dysfunction	1 (3.3)	1 (4)

**Fig. 1:**
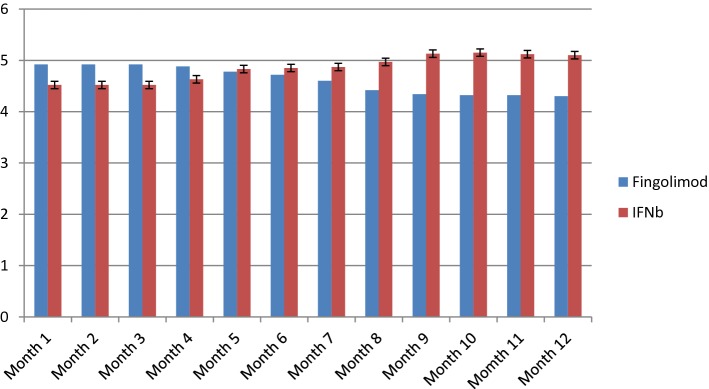
The EDSS scores in each month during the study period

Disease duration was 8.2 ± 3.42 and 8.77 ± 2.5 in treatment and control groups respectively (t = 0.7, *df* = 53, P = 0.48). EDSS scores were assessed in all patients each month (Table 3). Based on the results of Kolmogorov–Smirnov test regarding normal distribution of data, independent T test was used for comparison of mean values between two groups. EDSS scores were not different between two groups at the start point of the study and until month 7. However, EDSS scores were significantly lower in the patients treated with fingolimod from month 8 till the end of the study (P values of 0.004, 0.004, 0.006 and 0.007 respectively). Figure 1 shows the EDSS scores in each month during the study period.

**Table 3:** Number of relapses in each study group during the 12-month study period

Number of relapses	Study groups	
	Treatment group Number (%)	Control group Number (%)
0	25 (100)	0 (0)
1	0 (0)	11 (36.7)
2	0 (0)	19 (63.3)

Then, we compared EDSS scores in each study group in each time intervals. EDSS score in month 12 was significantly different from EDSS scores of month 1 to month 8 in fingolimod treated patients. In control group, significant differences were detected between EDSS score of month 12 and those of months 1–7.

We also assessed relapse rate in both study groups during the 12-month study period using Cochran–Armitage test (Table 3). Significant difference was detected in the number of relapses between two groups (P < 0.001).

Pearson correlation test showed significant correlation between EDSS in month 12 and patients’ age in treatment group (r = 0.45, P = 0.02). However, no significant correlation was found between these parameters in control group (r = 0.28, P = 0.13). No significant correlation was found between age and number of relapses in either groups (P > 0.05). No significant correlation was found between the number of relapses and EDSS score in the control group. We also compared mean EDSS scores in month 12 between males and females and found no difference in either groups (P = 0.47 and 0.54 in treatment and control groups respectively). No significant association was found between number of relapses and sex of patients in either groups (P > 0.05).

## Discussion

In the present non-randomized clinical trial, we assessed the effectiveness of oral fingolimod on relapse rate and disability progression in Iranian MS patients and found its effectiveness on improvement of both parameters. The effect of fingolimod on the annualized relapse rate, the amount of new or enlarged lesions on T(2)-weighted MRI and progression of disability has been assessed previously in a 12-month, double-blind, double-dummy, randomized clinical trial in which patients were randomly allocated to take either oral fingolimod at a daily dose of either 1.25 or 0.5 mg or intramuscular IFNβ-1a at a weekly dose of 30 µg. Both administered doses of fingolimod have been effective in decreasing the number of relapses but not disability score [5]. In two other double-blind, randomized study, the effectiveness of the mentioned doses of fingolimod was compared with placebo [5, 8]. Both studies reported the effectiveness of this drug on reduction of relapse rate, but the results were inconsistent regarding the disability progression [5, 8].

Notably, none of the patients treated with fingolimod in our current study experienced relapse during the 12-month study period which indicates appropriateness of this treatment strategy in Iranian patients. Based on the heterogeneity of MS in terms of disease course and response to treatments [9], population-based studies are needed to appraise effectiveness of each treatment strategy in distinct populations. Our obtained results are in line with the results of a retrospective study in Portuguese patients with RRMS which reported effectiveness of fingolimod in reduction of relapse rate and EDSS score after first-line treatment failure [10].

We also detected significant correlation between EDSS in month 12 and patients’ age in patients treated with fingolimod which is consistent with the previously reported role for age as an indispensable modifier of a drug efficacy [11]. Higher age has also been associated with reduced brain volume and EDSS score in FREEDOMS, FREEDOMS II, and TRANSFORMS phase III trials of fingolimod [12].

## Conclusion

In spite of safety concerns about administration of fingolimod, we did not detect any serious side effects possibly due to application of serious criteria for selection of patients. Finally, our study has some limitations especially in terms of sample size. Further assessment of fingolimod effect on patients’ disability based on MSSS (MS Severity Score) [13] is also suggested for estimation of disease severity over time.

## Abbreviations

MS: multiple sclerosis
EDSS: expanded disability status scale
RRMS: relapsing-remitting
S1P: sphingosine-1-phosphate
CNS: central nervous system
IFNβ: interferon β
ECG: electrocardiogram
